# Supplementary material for: Longitudinal Transcription Profiling of Bladder Cancers Dictate the Response to BCG Treatment and Disease Progression
Source: Int J Mol Sci. 2023 Dec 21;25(1):144. doi: 10.3390/ijms25010144 (PMC10778820; doi:10.3390/ijms25010144)
Supplement: Supplementary file 1 [file ijms-25-00144-s001.zip › Supple_Fig1.pdf]

A

B

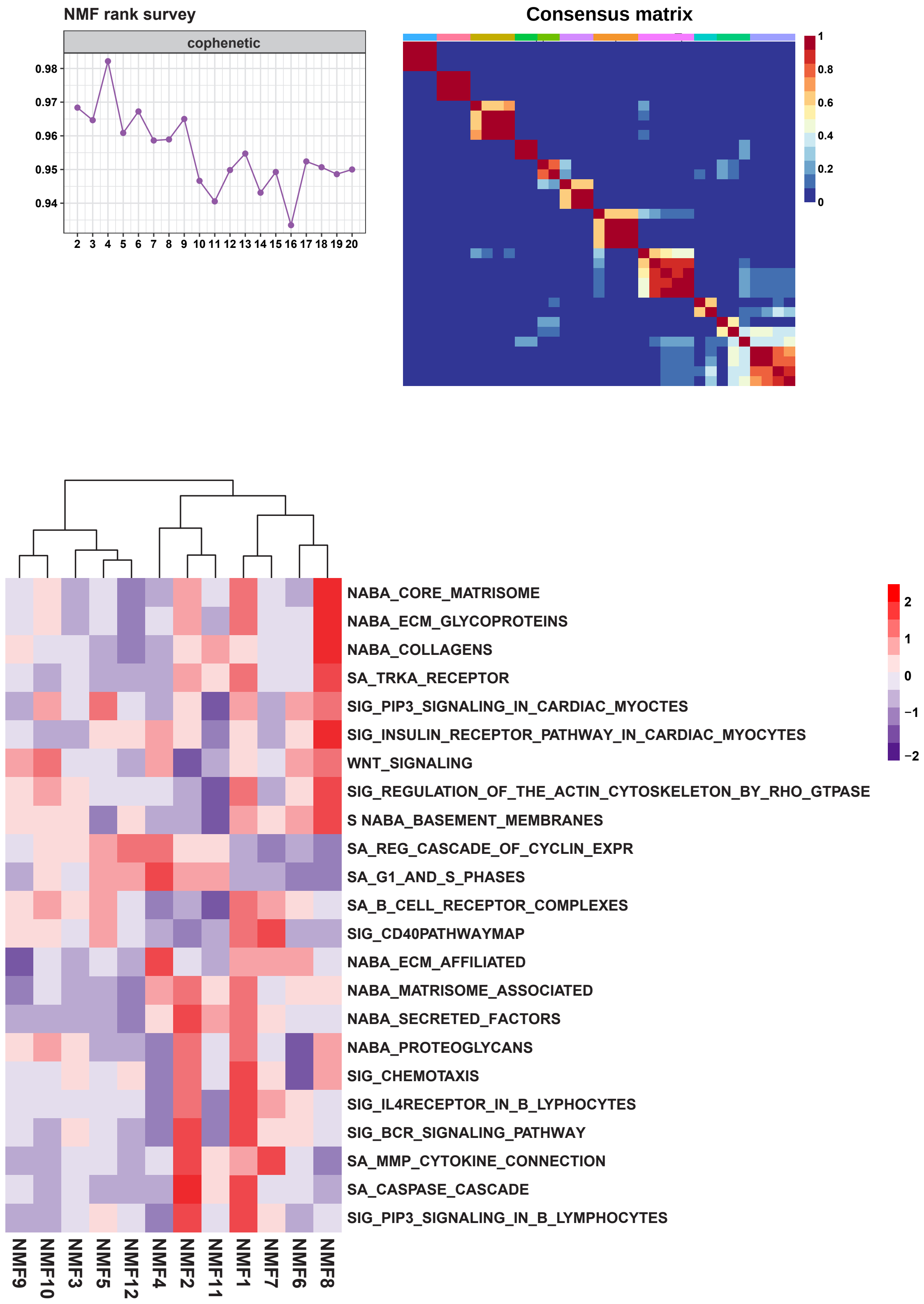
NMF rank survey
cophenetic
0.98
0.97
0.96
0.95
0.94
2 3 4 5 6 7 8 9 10 11 12 13 14 15 16 17 18 19 20
Consensus matrix
1
0.8
0.6
0.4
0.2
0
NABA_CORE_MATRISOME
NABA_ECM_GLYCOPROTEINS
NABA_COLLAGENS
SA_TRKA_RECEPTOR
SIG_PIP3_SIGNALING_IN_CARDIAC_MYOCTES
SIG_INSULIN_RECEPTOR_PATHWAY_IN_CARDIAC_MYOCYTES
WNT_SIGNALING
SIG_REGULATION_OF_THE_ACTIN_CYTOSKELETON_BY_RHO_GTPASE
S NABA_BASEMENT_MEMBRANES
SA_REG_CASCADE_OF_CYCLIN_EXPR
SA_G1_AND_S_PHASES
SA_B_CELL_RECEPTOR_COMPLEXES
SIG_CD40PATHWAYMAP
NABA_ECM_AFFILIATED
NABA_MATRISOME_ASSOCIATED
NABA_SECRETED_FACTORS
NABA_PROTEOGLYCANS
SIG_CHEMOTAXIS
SIG_IL4RECEPTOR_IN_B_LYPHOCYTES
SIG_BCR_SIGNALING_PATHWAY
SA_MMP_CYTOKINE_CONNECTION
SA_CASPASE_CASCADE
SIG_PIP3_SIGNALING_IN_B_LYMPHOCYTES
2
1
0
−1
−2
NMF9
NMF10
NMF3
NMF5
NMF12
NMF4
NMF2
NMF11
NMF1
NMF7
NMF6
NMF8

C

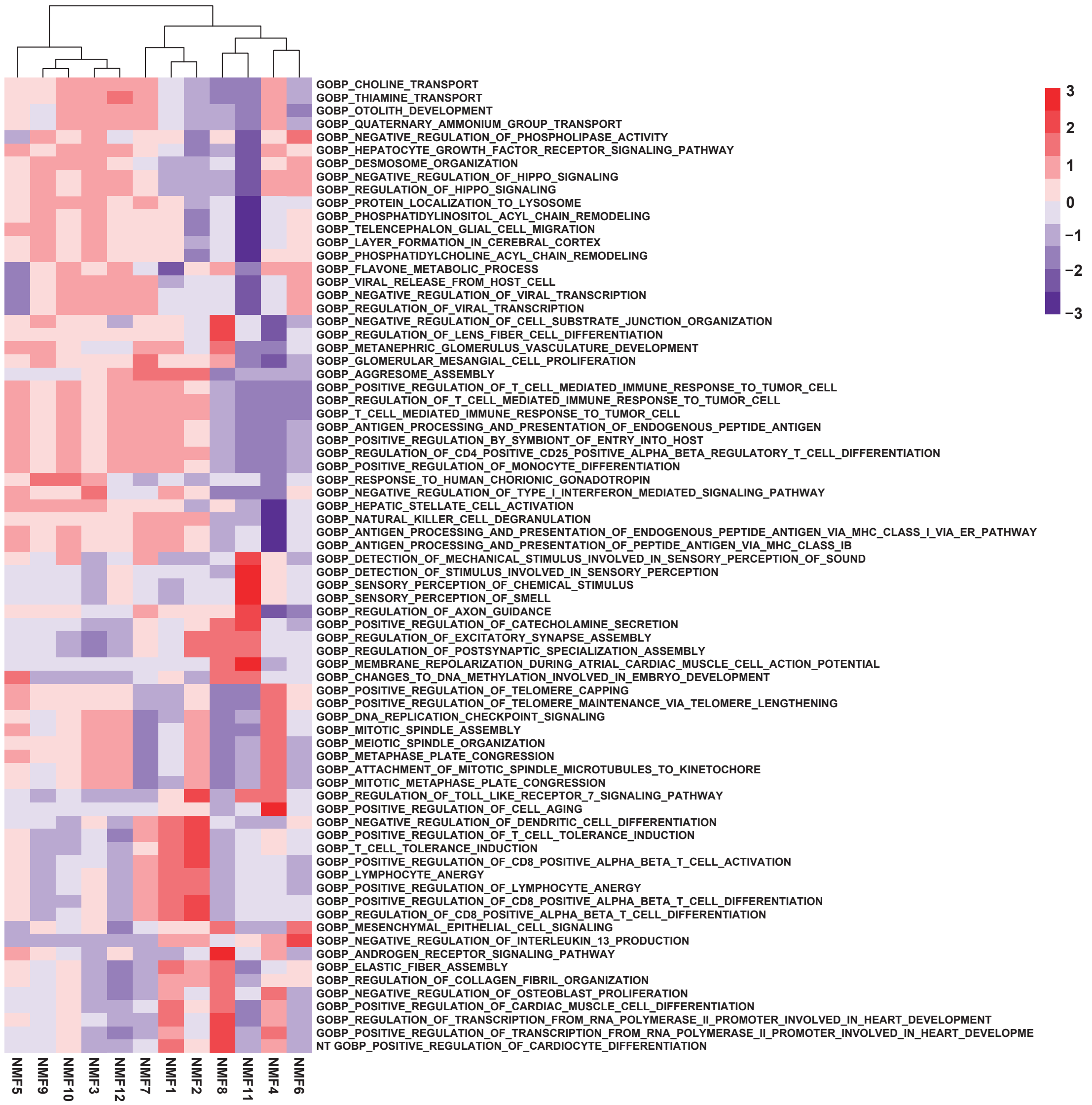
GOBP_CHOLINE_TRANSPORT
GOBP_THIAMINE_TRANSPORT
GOBP_OTOLITH_DEVELOPMENT
GOBP_QUATERNARY_AMMONIUM_GROUP_TRANSPORT
GOBP_NEGATIVE_REGULATION_OF_PHOSPHOLIPASE_ACTIVITY
GOBP_HEPATOCYTE_GROWTH_FACTOR_RECEPTOR_SIGNALING_PATHWAY
GOBP_DESMOSOME_ORGANIZATION
GOBP_NEGATIVE_REGULATION_OF_HIPPO_SIGNALING
GOBP_REGULATION_OF_HIPPO_SIGNALING
GOBP_PROTEIN_LOCALIZATION_TO_LYSOSOME
GOBP_PHOSPHATIDYLINOSITOL_ACYL_CHAIN_REMODELING
GOBP_TELENCEPHALON_GLIAL_CELL_MIGRATION
GOBP_LAYER_FORMATION_IN_CEREBRAL_CORTEX
GOBP_PHOSPHATIDYLCHOLINE_ACYL_CHAIN_REMODELING
GOBP_FLAVONE_METABOLIC_PROCESS
GOBP_VIRAL_RELEASE_FROM_HOST_CELL
GOBP_NEGATIVE_REGULATION_OF_VIRAL_TRANSCRIPTION
GOBP_REGULATION_OF_VIRAL_TRANSCRIPTION
GOBP_NEGATIVE_REGULATION_OF_CELL_SUBSTRATE_JUNCTION_ORGANIZATION
GOBP_REGULATION_OF_LENS_FIBER_CELL_DIFFERENTIATION
GOBP_METANEPHRIC_GLOMERULUS_VASCULATURE_DEVELOPMENT
GOBP_GLOMERULAR_MESANGIAL_CELL_PROLIFERATION
GOBP_AGGRESOME_ASSEMBLY
GOBP_POSITIVE_REGULATION_OF_T_CELL_MEDIATED_IMMUNE_RESPONSE_TO_TUMOR_CELL
GOBP_REGULATION_OF_T_CELL_MEDIATED_IMMUNE_RESPONSE_TO_TUMOR_CELL
GOBP_T_CELL_MEDIATED_IMMUNE_RESPONSE_TO_TUMOR_CELL
GOBP_ANTIGEN_PROCESSING_AND_PRESENTATION_OF_ENDOGENOUS_PEPTIDE_ANTIGEN
GOBP_POSITIVE_REGULATION_BY_SYMBIONT_OF_ENTRY_INTO_HOST
GOBP_REGULATION_OF_CD4_POSITIVE_CD25_POSITIVE_ALPHA_BETA_REGULATORY_T_CELL_DIFFERENTIATION
GOBP_POSITIVE_REGULATION_OF_MONOCYTE_DIFFERENTIATION
GOBP_RESPONSE_TO_HUMAN_CHORIONIC_GONADOTROPIN
GOBP_NEGATIVE_REGULATION_OF_TYPE_I_INTERFERON_MEDIATED_SIGNALING_PATHWAY
GOBP_HEPATIC_STELLATE_CELL_ACTIVATION
GOBP_NATURAL_KILLER_CELL_DEGRANULATION
GOBP_ANTIGEN_PROCESSING_AND_PRESENTATION_OF_ENDOGENOUS_PEPTIDE_ANTIGEN_VIA_MHC_CLASS_I_VIA_ER_PATHWAY
GOBP_ANTIGEN_PROCESSING_AND_PRESENTATION_OF_PEPTIDE_ANTIGEN_VIA_MHC_CLASS_IB
GOBP_DETECTION_OF_MECHANICAL_STIMULUS_INVOLVED_IN_SENSORY_PERCEPTION_OF_SOUND
GOBP_DETECTION_OF_STIMULUS_INVOLVED_IN_SENSORY_PERCEPTION
GOBP_SENSORY_PERCEPTION_OF_CHEMICAL_STIMULUS
GOBP_SENSORY_PERCEPTION_OF_SMELL
GOBP_REGULATION_OF_AXON_GUIDANCE
GOBP_POSITIVE_REGULATION_OF_CATECHOLAMINE_SECRETION
GOBP_REGULATION_OF_EXCITATORY_SYNAPSE_ASSEMBLY
GOBP_REGULATION_OF_POSTSYNAPTIC_SPECIALIZATION_ASSEMBLY
GOBP_MEMBRANE_REPOLARIZATION_DURING_ATRIAL_CARDIAC_MUSCLE_CELL_ACTION_POTENTIAL
GOBP_CHANGES_TO_DNA_METHYLATION_INVOLVED_IN_EMBRYO_DEVELOPMENT
GOBP_POSITIVE_REGULATION_OF_TELOMERE_CAPPING
GOBP_POSITIVE_REGULATION_OF_TELOMERE_MAINTENANCE_VIA_TELOMERE_LENGTHENING
GOBP_DNA_REPLICATION_CHECKPOINT_SIGNALING
GOBP_MITOTIC_SPINDLE_ASSEMBLY
GOBP_MEIOTIC_SPINDLE_ORGANIZATION
GOBP_METAPHASE_PLATE_CONGRESSION
GOBP_ATTACHMENT_OF_MITOTIC_SPINDLE_MICROTUBULES_TO_KINETOCHORE
GOBP_MITOTIC_METAPHASE_PLATE_CONGRESSION
GOBP_REGULATION_OF_TOLL_LIKE_RECEPTOR_7_SIGNALING_PATHWAY
GOBP_POSITIVE_REGULATION_OF_CELL_AGING
GOBP_NEGATIVE_REGULATION_OF_DENDRITIC_CELL_DIFFERENTIATION
GOBP_POSITIVE_REGULATION_OF_T_CELL_TOLERANCE_INDUCTION
GOBP_T_CELL_TOLERANCE_INDUCTION
GOBP_POSITIVE_REGULATION_OF_CD8_POSITIVE_ALPHA_BETA_T_CELL_ACTIVATION
GOBP_LYMPHOCYTE_ANERGY
GOBP_POSITIVE_REGULATION_OF_LYMPHOCYTE_ANERGY
GOBP_POSITIVE_REGULATION_OF_CD8_POSITIVE_ALPHA_BETA_T_CELL_DIFFERENTIATION
GOBP_REGULATION_OF_CD8_POSITIVE_ALPHA_BETA_T_CELL_DIFFERENTIATION
GOBP_MESENCHYMAL_EPITHELIAL_CELL_SIGNALING
GOBP_NEGATIVE_REGULATION_OF_INTERLEUKIN_13_PRODUCTION
GOBP_ANDROGEN_RECEPTOR_SIGNALING_PATHWAY
GOBP_ELASTIC_FIBER_ASSEMBLY
GOBP_REGULATION_OF_COLLAGEN_FIBRIL_ORGANIZATION
GOBP_NEGATIVE_REGULATION_OF_OSTEOBLAST_PROLIFERATION
GOBP_POSITIVE_REGULATION_OF_CARDIAC_MUSCLE_CELL_DIFFERENTIATION
GOBP_REGULATION_OF_TRANSCRIPTION_FROM_RNA_POLYMERASE_II_PROMOTER_INVOLVED_IN_HEART_DEVELOPMENT
GOBP_POSITIVE_REGULATION_OF_TRANSCRIPTION_FROM_RNA_POLYMERASE_II_PROMOTER_INVOLVED_IN_HEART_DEVELOPME
NT GOBP_POSITIVE_REGULATION_OF_CARDIOCYTE_DIFFERENTIATION
NMF5
NMF9
NMF10
NMF3
NMF12
NMF7
NMF1
NMF2
NMF8
NMF11
NMF4
NMF6
3
2
1
0
−1
−2
−3

D

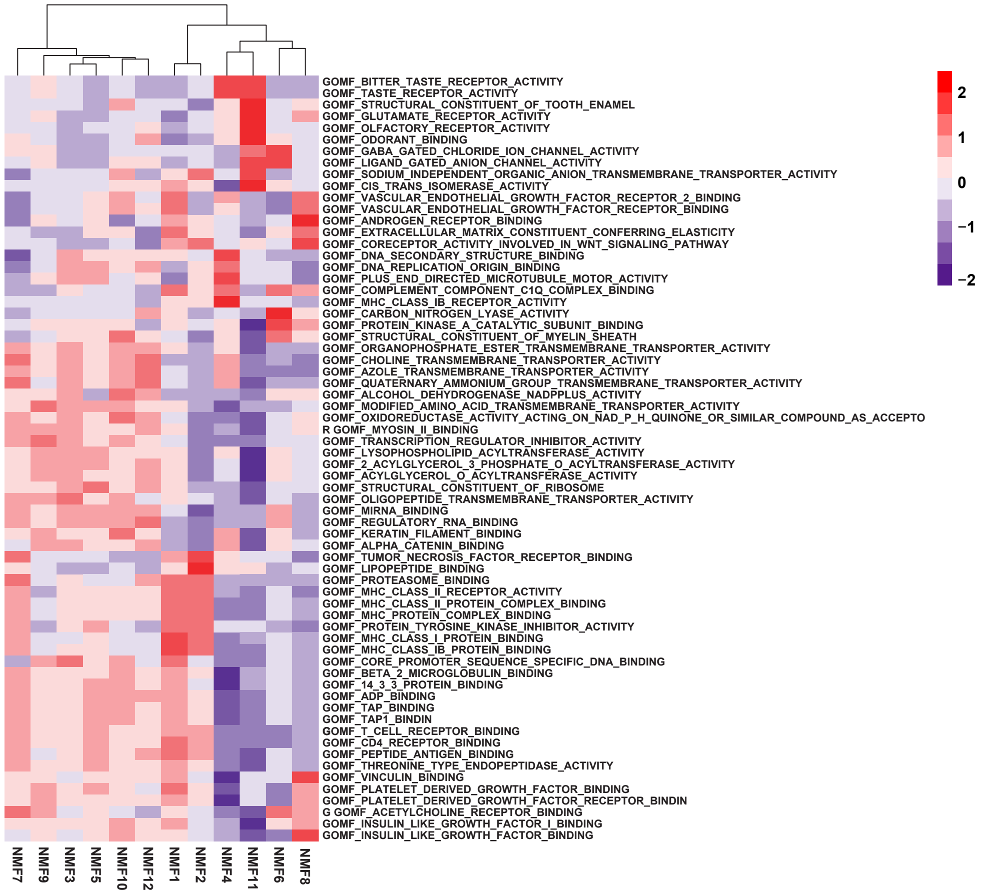
GOMF_BITTER_TASTE_RECEPTOR_ACTIVITY
GOMF_TASTE_RECEPTOR_ACTIVITY
GOMF_STRUCTURAL_CONSTITUENT_OF_TOOTH_ENAMEL
GOMF_GLUTAMATE_RECEPTOR_ACTIVITY
GOMF_OLFACTORY_RECEPTOR_ACTIVITY
GOMF_ODORANT_BINDING
GOMF_GABA_GATED_CHLORIDE_ION_CHANNEL_ACTIVITY
GOMF_LIGAND_GATED_ANION_CHANNEL_ACTIVITY
GOMF_SODIUM_INDEPENDENT_ORGANIC_ANION_TRANSMEMBRANE_TRANSPORTER_ACTIVITY
GOMF_CIS_TRANS_ISOMERASE_ACTIVITY
GOMF_VASCULAR_ENDOTHELIAL_GROWTH_FACTOR_RECEPTOR_2_BINDING
GOMF_VASCULAR_ENDOTHELIAL_GROWTH_FACTOR_RECEPTOR_BINDING
GOMF_ANDROGEN_RECEPTOR_BINDING
GOMF_EXTRACELLULAR_MATRIX_CONSTITUENT_CONFERRING_ELASTICITY
GOMF_CORECEPTOR_ACTIVITY_INVOLVED_IN_WNT_SIGNALING_PATHWAY
GOMF_DNA_SECONDARY_STRUCTURE_BINDING
GOMF_DNA_REPLICATION_ORIGIN_BINDING
GOMF_PLUS_END_DIRECTED_MICROTUBULE_MOTOR_ACTIVITY
GOMF_COMPLEMENT_COMPONENT_C1Q_COMPLEX_BINDING
GOMF_MHC_CLASS_IB_RECEPTOR_ACTIVITY
GOMF_CARBON_NITROGEN_LYASE_ACTIVITY
GOMF_PROTEIN_KINASE_A_CATALYTIC_SUBUNIT_BINDING
GOMF_STRUCTURAL_CONSTITUENT_OF_MYELIN_SHEATH
GOMF_ORGANOPHOSPHATE_ESTER_TRANSMEMBRANE_TRANSPORTER_ACTIVITY
GOMF_CHOLINE_TRANSMEMBRANE_TRANSPORTER_ACTIVITY
GOMF_AZOLE_TRANSMEMBRANE_TRANSPORTER_ACTIVITY
GOMF_QUATERNARY_AMMONIUM_GROUP_TRANSMEMBRANE_TRANSPORTER_ACTIVITY
GOMF_ALCOHOL_DEHYDROGENASE_NADPPLUS_ACTIVITY
GOMF_MODIFIED_AMINO_ACID_TRANSMEMBRANE_TRANSPORTER_ACTIVITY
GOMF_OXIDOREDUCTASE_ACTIVITY_ACTING_ON_NAD_P_H_QUINONE_OR_SIMILAR_COMPOUND_AS_ACCEPTO
R GOMF_MYOSIN_II_BINDING
GOMF_TRANSCRIPTION_REGULATOR_INHIBITOR_ACTIVITY
GOMF_LYSOPHOSPHOLIPID_ACYLTRANSFERASE_ACTIVITY
GOMF_2_ACYLGLYCEROL_3_PHOSPHATE_O_ACYLTRANSFERASE_ACTIVITY
GOMF_ACYLGLYCEROL_O_ACYLTRANSFERASE_ACTIVITY
GOMF_STRUCTURAL_CONSTITUENT_OF_RIBOSOME
GOMF_OLIGOPEPTIDE_TRANSMEMBRANE_TRANSPORTER_ACTIVITY
GOMF_MIRNA_BINDING
GOMF_REGULATORY_RNA_BINDING
GOMF_KERATIN_FILAMENT_BINDING
GOMF_ALPHA_CATENIN_BINDING
GOMF_TUMOR_NECROSIS_FACTOR_RECEPTOR_BINDING
GOMF_LIPOPEPTIDE_BINDING
GOMF_PROTEASOME_BINDING
GOMF_MHC_CLASS_II_RECEPTOR_ACTIVITY
GOMF_MHC_CLASS_II_PROTEIN_COMPLEX_BINDING
GOMF_MHC_PROTEIN_COMPLEX_BINDING
GOMF_PROTEIN_TYROSINE_KINASE_INHIBITOR_ACTIVITY
GOMF_MHC_CLASS_I_PROTEIN_BINDING
GOMF_MHC_CLASS_IB_PROTEIN_BINDING
GOMF_CORE_PROMOTER_SEQUENCE_SPECIFIC_DNA_BINDING
GOMF_BETA_2_MICROGLOBULIN_BINDING
GOMF_14_3_3_PROTEIN_BINDING
GOMF_ADP_BINDING
GOMF_TAP_BINDING
GOMF_TAP1_BINDIN
GOMF_T_CELL_RECEPTOR_BINDING
GOMF_CD4_RECEPTOR_BINDING
GOMF_PEPTIDE_ANTIGEN_BINDING
GOMF_THREONINE_TYPE_ENDOPEPTIDASE_ACTIVITY
GOMF_VINCULIN_BINDING
GOMF_PLATELET_DERIVED_GROWTH_FACTOR_BINDING
GOMF_PLATELET_DERIVED_GROWTH_FACTOR_RECEPTOR_BINDIN
G GOMF_ACETYLCHOLINE_RECEPTOR_BINDING
GOMF_INSULIN_LIKE_GROWTH_FACTOR_I_BINDING
GOMF_INSULIN_LIKE_GROWTH_FACTOR_BINDING
NMF7
NMF9
NMF3
NMF5
NMF10
NMF12
NMF1
NMF2
NMF4
NMF11
NMF6
NMF8
2
1
0
−1
−2

E

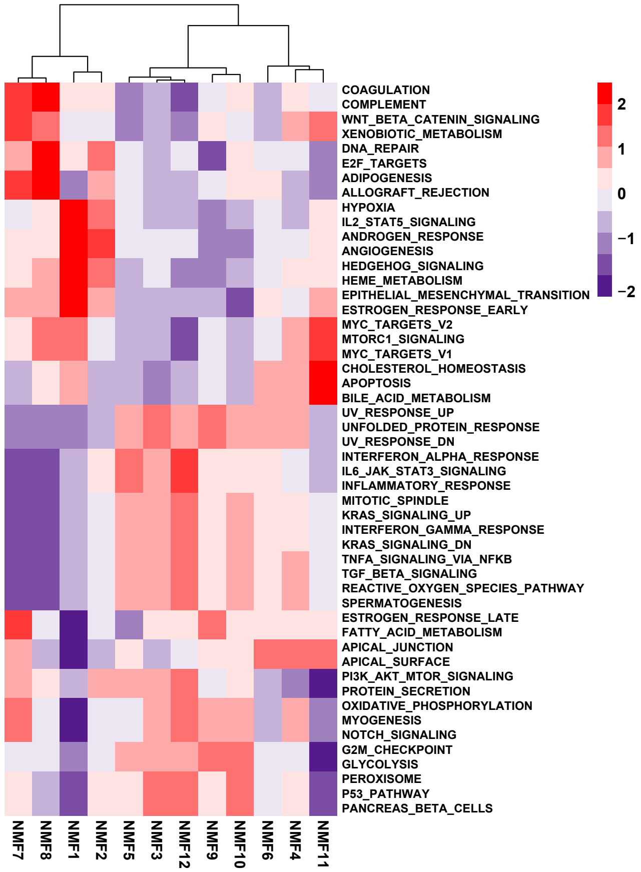

Figure S1. Analysis of pre- and post-treatment datasets using NMF. A, Cophenetic distribution was calculated in 2-20 runs. We selected K=12 as optimal and visualized the consensus matrix to calculate the robustness. B, Heatmap of the Curated gene sets pathway result. C, Heatmap of the Ontology gene sets (BP) pathway result. D, Heatmap of the Ontology gene sets (MF) pathway result. E, Heatmap of the Hallmark gene sets pathway result.
