# Supplementary material for: Longitudinal Transcription Profiling of Bladder Cancers Dictate the Response to BCG Treatment and Disease Progression
Source: Int J Mol Sci. 2023 Dec 21;25(1):144. doi: 10.3390/ijms25010144 (PMC10778820; doi:10.3390/ijms25010144)
Supplement: Supplementary file 1 [file ijms-25-00144-s001.zip › Supple_Fig2.pdf]

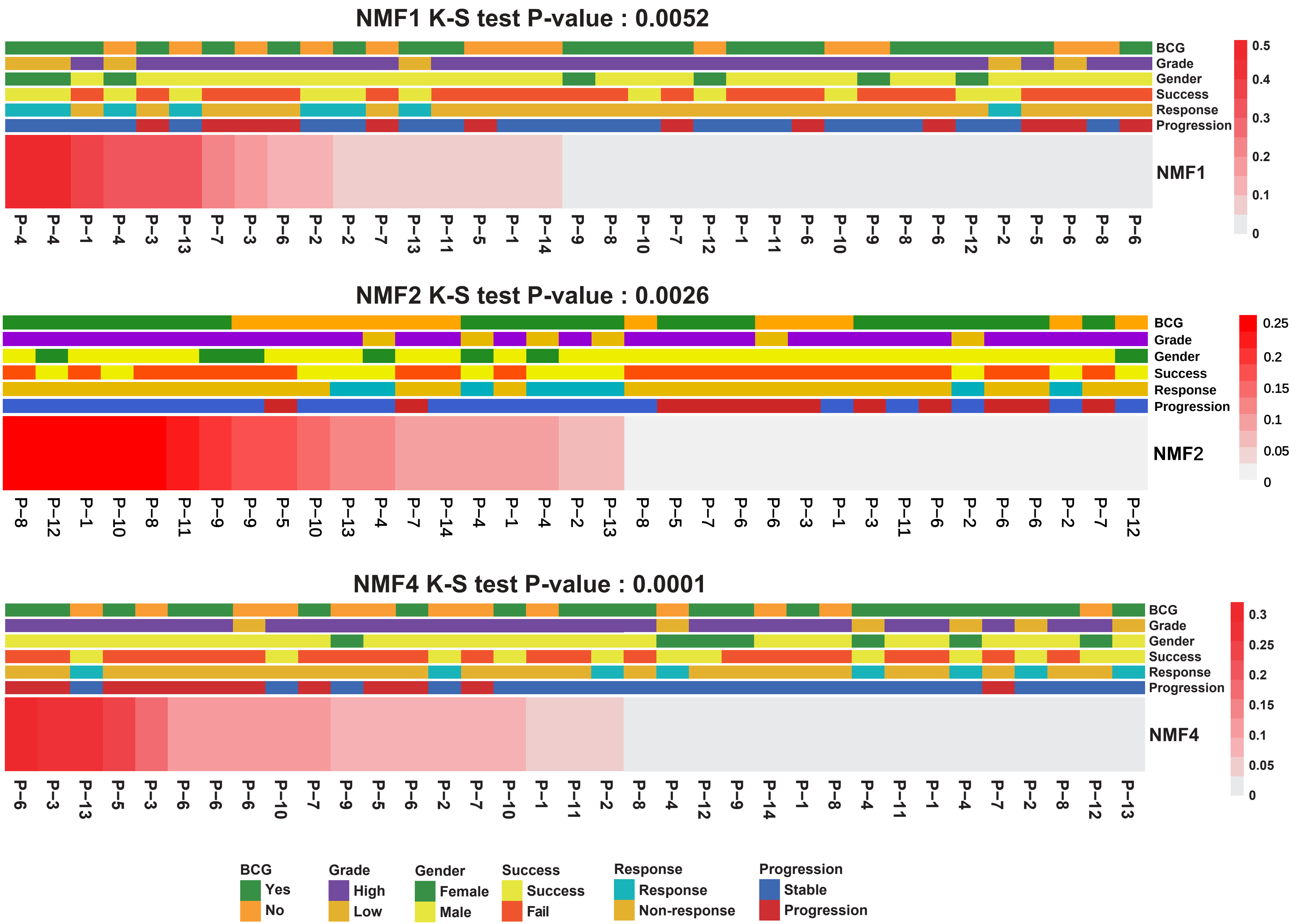

Figure S2. Visualization coefficients of NMF1, NMF2 and NMF4. A, Ordering the NMF1, NMF2, and NMF4 coefficients with the clinical course
